# Evaluation of Third‐Order Motion‐Compensated Cardiac Diffusion Tensor Imaging Across Cardiac Phases Using an Ultra‐High‐Performance Clinical Scanner

**DOI:** 10.1002/mrm.70395

**Published:** 2026-04-22

**Authors:** Ke Wen, Pedro F. Ferreira, Alberto Di Biase Oemick, Ricardo Wage, Karl P. Kunze, Fanwen Wang, Dudley J. Pennell, Andrew D. Scott, Sonia Nielles‐Vallespin

**Affiliations:** ^1^ National Heart and Lung Institute Imperial College London London UK; ^2^ Cardiovascular Magnetic Resonance Unit Royal Brompton Hospital, Guy's and St Thomas' NHS Foundation Trust London UK; ^3^ EPSRC Centre for Doctoral Training in Smart Medical Imaging King's College London and Imperial College London London UK; ^4^ Department of Computing Imperial College London London UK; ^5^ MR Research Collaborations Siemens Healthcare Limited Camberley UK; ^6^ Department of Bioengineering Imperial College London London UK

**Keywords:** cardiac DTI, motion compensation, ultra‐high‐performance gradient

## Abstract

**Purpose:**

To evaluate a third‐order motion‐compensated spin echo (M3‐MCSE) sequence at multiple cardiac phases on a clinical 3 T MRI scanner with ultra‐high performance (UHP) gradients (200 mT/m), compared with stimulated echo acquisition mode (STEAM) and second‐order MCSE (M2‐MCSE) for cardiac diffusion tensor imaging (cDTI).

**Methods:**

Twenty healthy subjects underwent mid‐ventricular short‐axis cDTI at peak systole and diastasis using STEAM, M2‐MCSE, and M3‐MCSE. cDTI metrics and image quality were compared. In five additional healthy subjects, diffusion‐weighted images were obtained at multiple trigger delays distributed over diastasis to assess motion‐induced signal loss.

**Results:**

Compared to M2‐MCSE, M3‐MCSE yielded higher systolic helix angle map scores (p=0.007) but lower diastolic scores (p=0.001), with no significant difference in mean diffusivity, fractional anisotropy, helix angle transmurality or sheetlet angle in systole/diastole. STEAM‐derived apparent diffusion coefficients (ADC) were consistent across diastasis, while ADC for MCSE sequences increased at sub‐optimal trigger delays.

**Conclusion:**

UHP gradients enabled in vivo evaluation of M3‐MCSE, showing superior systolic cDTI but reduced diastolic performance versus M2‐MCSE due to reduced signal‐to‐noise ratio and a longer motion‐sensitive window. Future work may consider numerically optimized gradient designs to enhance MCSE robustness throughout the cardiac cycle.

## 1 Introduction

Cardiac diffusion tensor imaging (cDTI) is an innovative technology that allows noninvasive in vivo characterization of myocardial microstructural dynamics. It has provided valuable and novel insights into various cardiac diseases [1, 2, 3, 4, 5, 6]. cDTI utilizes diffusion‐sensitive pulse sequence to quantify water molecule displacement and depicts tissue microstructure by measuring the signal loss due to the incoherent motion of spins during diffusion encoding gradients designed to impart a signal phase that is linear with displacement.

**FIGURE 1:**
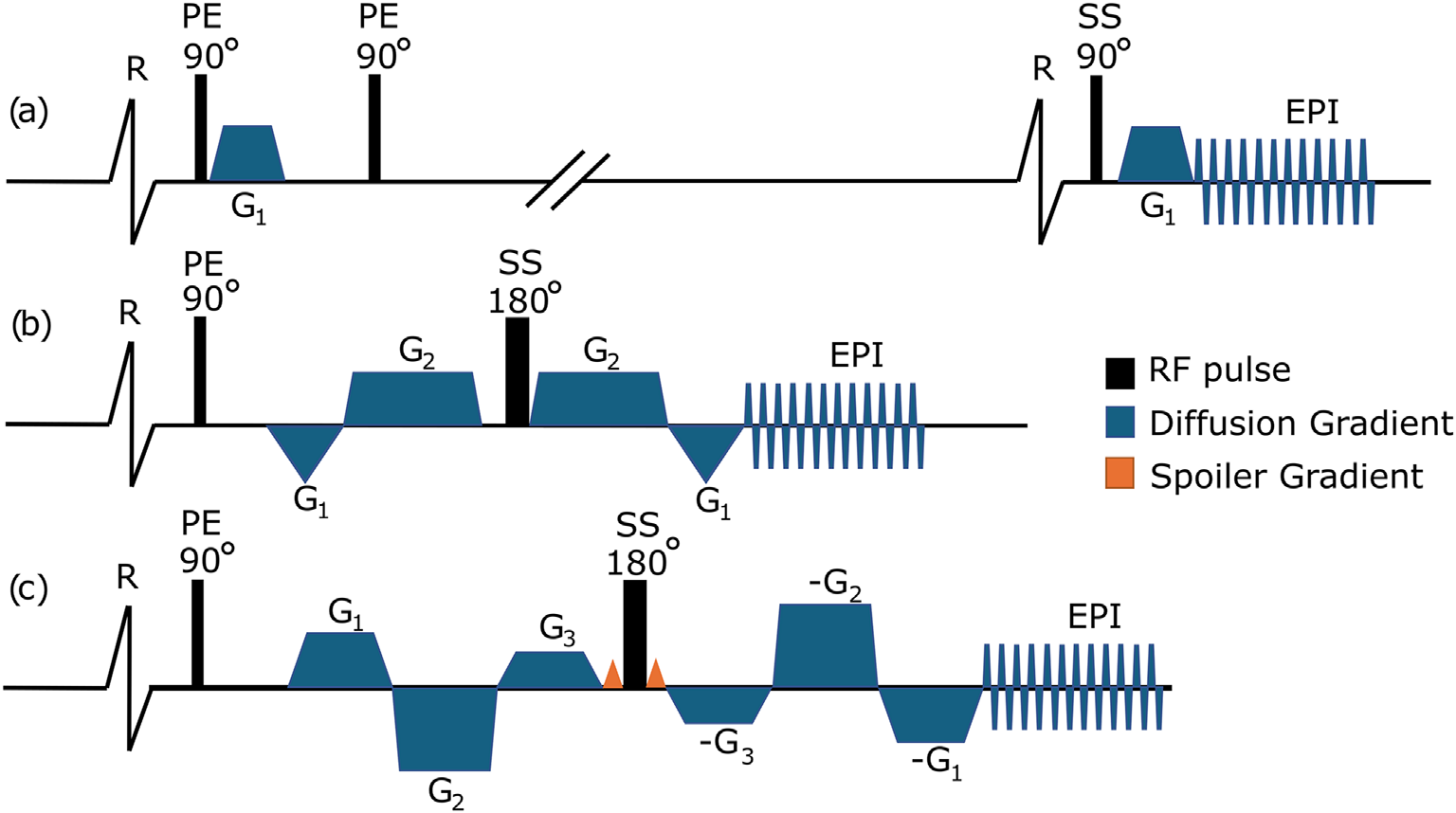
STEAM (a), M2‐MCSE (b) and M3‐MCSE (c) sequences with matched EPI readouts. M3‐MCSE, with its current design, requires spoiler gradients to create a finite zeroth‐order gradient moment when the refocusing RF pulse is applied to spoil free‐induction signals caused by deviations from the ideal 180° flip angle. Zonal excitation is applied by modifying the first two RF pulses for STEAM and the excitation pulse for MCSEs to make them slice‐selective in the phase‐encoding (PE) direction, keeping the last RF pulses in the slice‐selection (SS) direction.

The stimulated echo acquisition mode (STEAM) sequence is well‐established for cDTI [7, 8] and has been shown to be reliable in both systole and diastole. It achieves diffusion encoding by spanning the acquisition process over two consecutive heartbeats (Figure 1), resulting in a diffusion time (Δ) of an entire cardiac cycle (R–R interval). Consequently, the diffusion encoding gradients for STEAM can have relatively low amplitude and short duration while achieving high diffusion sensitivity (*b*‐value). While STEAM has low gradient hardware requirements, it is hindered by the assumption that the two consecutive heartbeats are identical and is intrinsically signal‐to‐noise ratio (SNR) inefficient [5, 9].

Motion‐compensated spin echo (MCSE) sequences [9, 10, 11, 12] can potentially overcome these issues by acquiring diffusion‐weighted (DW) images within a single cardiac cycle, providing higher SNR efficiency. MCSE sequences apply diffusion encoding through delicately designed motion‐compensated gradient waveforms, enabling the predictable phase accumulation from bulk motion to be nulled to a specific order, reducing motion‐related signal loss.

The first‐order motion MCSE (M1‐MCSE), which compensates for motion with constant velocity, has been shown to be effective only within a limited systolic window [10, 11], and remains inadequate for clinical translation. To address this, second‐order MCSE (M2‐MCSE) sequences [5, 9, 11, 13, 14, 15] have been proposed, where the phase accumulation due to constant velocity and constant acceleration during the diffusion encoding gradients is nulled.

While M2‐MCSE demonstrates robust systolic performance, challenges remain for diastole. Two studies have reported high failure rates with diastolic cDTI using M2‐MCSE [5, 13], while other studies suggested that imaging in mid‐diastole with M2‐MCSE requires careful planning based on cine and phase‐contrast quantitative flow imaging [16, 17], limiting its clinical applicability. These studies suggest that the complexity of diastolic cardiac motion beyond constant acceleration may lead to inadequate motion compensation.

Third‐order motion‐compensated spin echo (M3‐MCSE) [15], which incorporates additional compensation for constant jerk (the rate of change of acceleration), may provide improved robustness to cardiac motion and potentially address this issue. While a limited number of studies have evaluated in vivo M3‐MCSE cDTI during systole [15, 18], its potential in diastole remains unexplored.

A key challenge is that M3‐MCSE requires more complex gradient waveforms, which extend the echo time (TE), resulting in increased T2‐related signal loss and a longer motion‐sensitive duration. Recent advancements in commercial scanners equipped with ultra‐high‐performance (UHP) gradient systems (200 mT/m, 200 T/m/s) provide new opportunities for cDTI in two key ways: (1) by shortening motion‐sensitive diffusion encoding duration, improving the performance of first‐ or second‐order motion compensation; (2) by enabling higher motion compensation while maintaining reasonable TE.

Kara et al. [14] evaluated zeroth‐, first‐, and second‐order motion compensation with a UHP gradient system and demonstrated that second‐order motion compensation remains essential to mitigate motion‐related signal loss. However, their evaluation remains limited to systole, and distolic M2‐MCSE using a UHP gradient has not been previously assessed. Whether UHP would facilitate more robust diastolic M2‐MCSE remains unclear. Furthermore, the commercial UHP gradient system enables the practical implementation of M3‐MCSE despite the extended diffusion encoding gradient waveform, offering a potential solution for robust diastolic imaging. However, for in vivo applications, the effective gradient amplitude and slew rate may be constrained by safety considerations such as peripheral nerve stimulation (PNS) and cardiac stimulation [18].

This study aims to address these gaps by systematically evaluating higher‐order motion compensation M3‐MCSE on a commercially available scanner with whole‐body UHP gradients, and compare it with STEAM and M2‐MCSE in both systole and diastole, with a particular focus on assessing motion‐related signal loss during diastole.

## 2 Methods

Twenty‐two healthy volunteers (Table S1) with no known cardiac disease were recruited with informed consent under local ethical approval. Twenty completed the full cDTI acquisition at both peak systole and diastasis. Five volunteers underwent a separate scan for the motion robustness sub‐study, three of whom also participated in the main cDTI scans in separate sessions.

STEAM, M2‐MCSE, and M3‐MCSE (Figure 1) were implemented on a commercial 3 T scanner with a UHP gradient system (MAGNETOM Cima. X, Siemens Healthineers, Forchheim, Germany, 200 mT/m and 200 T/m/s), with STEAM and M2‐MCSE as described in previous studies [13]. While M2‐MCSE used symmetric trapezoidal diffusion‐encoding waveforms with constant amplitude [11], the M3‐MCSE sequence had an anti‐symmetric diffusion‐encoding waveform using trapezoidal gradient lobes with constant duration as described by Welsh et al. [15]. Spoiler gradients were required in M3‐MCSE to prevent nulled zeroth gradient moment at the 180° pulse to account for imperfect refocusing and, therefore, avoid gradient echo contamination in the measured images. The diffusion encoding gradients achieved a maximum amplitude of 144 mT/m and a maximum effective slew rate of 37 T/m/s among all three sequences (Table S2).

### 2.1 Study Protocol 1: cDTI at Peak Systole and Diastasis

A short‐axis mid‐ventricular cine acquisition was acquired to identify suitable trigger delays (TDs) for peak systole and diastasis. Acquisition protocols were closely matched between cDTI sequences. The readout field‐of‐view (FOV) of all DW protocols was $360\times 135\;{\mathrm{mm}}^{2}$, with zonal excitation [19] applied to reduce the FOV in the phase‐encoding (PE) direction. The resolution of DW images was $2.8\times 2.8\times 8\;{\mathrm{mm}}^{3}$ (interpolated to $1.4\times 1.4\times 8\;{\mathrm{mm}}^{3}$ in the vendor‐supplied reconstruction). For STEAM, the first two 90° pulses were slice selective in the PE direction, and the third 90° pulse in the slice‐selection (SS) direction. For both MCSE acquisitions, the RF excitation 90° pulse was selective in the PE direction, and the refocusing 180° pulse was selective in the SS direction. The echo‐planar imaging (EPI) readout was identical for all three sequences, with a parallel imaging factor of 2 applied (SENSE × 2) and a resultant acquired echo train length of 23. TE was minimized for each sequence, resulting in 24, 51, and 74 ms for STEAM, M2‐MCSE, and M3‐MCSE, respectively. The repetition time (TR) used for all three sequences was 2 RR intervals. Chemical shift selective fat saturation [20] was applied.

The TDs for the M2‐MCSE sequence were determined such that the center of the diffusion encoding gradients was at either peak systole or the visually most stationary period of diastasis based on cine imaging. For STEAM and M3‐MCSE, the TDs were adjusted so the midpoint of the diffusion encoding occurs at the same cardiac timing as in the M2‐MCSE acquisition [9]. Each sequence required 10 breath‐holds (18 heartbeats per breath‐hold) for each cardiac phase. Before the cDTI acquisition, the image orientation and PE direction were adjusted to displace the fat artifacts away from the myocardium. These adjustments were made to optimize systolic M2‐MCSE and not repeated for M3‐MCSE or STEAM, whose parameters were matched to M2‐MCSE.

Each cDTI dataset contained 10 repetitions of reference *b*‐value images ($b_{\mathrm{ref}}$ for STEAM = $34\;s/{\mathrm{mm}}^{2}$, for M2‐MCSE = $0.20\;s/{\mathrm{mm}}^{2}\approx 0\;s/{\mathrm{mm}}^{2}$, for M3‐MCSE = $0.16\;s/{\mathrm{mm}}^{2}\approx 0\;s/{\mathrm{mm}}^{2}$), 2 repetitions of low‐*b*‐value images sets ($b_{\mathrm{low}}$ for STEAM = $150\;s/{\mathrm{mm}}^{2}$, for M2‐MCSE = $50\;s/{\mathrm{mm}}^{2}$, for M3‐MCSE = $50\;s/{\mathrm{mm}}^{2}$, ×6directions), and 8 repetitions of high‐*b*‐value images sets ($b_{\text{high}}$ for STEAM = $600\;s/{\mathrm{mm}}^{2}$, for M2‐MCSE = $500\;s/{\mathrm{mm}}^{2}$, for M3‐MCSE = $500\;s/{\mathrm{mm}}^{2}$, ×6directions). For the STEAM sequence, the encoding assumed an RR interval of 1000 ms.

To disentangle the effect of motion from other confounding factors and to evaluate the eddy current effect, the same study was also performed on a stationary phantom without microscale structures, with an additional reference monopolar spin echo sequence following the same *b*‐value schemes as MCSEs (TE = 30 ms). The geometric distortion led by eddy current was quantified by calculating the pixel‐wise coefficient of variation (CoV) [21] across diffusion encoding directions for the $b_{\text{high}}$ images (Supporting Information).

### 2.2 Study Protocol 2: Evaluating Motion Robustness Throughout Diastasis

We evaluated the performance of the three sequences across diastasis for their robustness to motion in five healthy volunteers scanned separately. We identified the start and end of diastasis using the short‐axis cine image and captured 5–8 sets of DW images at various TDs during diastasis. To minimize mis‐triggering, TDs near the R wave were avoided, and the maximum attempted TD was set to 90% of the diastasis window. DW images were acquired with encoding in three orthogonal directions: phase, readout, and slice (PRS). Each DW dataset included 6 repetitions of the $b_{\mathrm{ref}}$ and $b_{\text{high}}$ images.

### 2.3 Data Analysis

cDTI datasets were post‐processed using the open‐source Python‐based cDTI analysis tool, INDI [22].

#### 2.3.1 Study Protocol 1

All cDTI datasets were post‐processed following our standard protocol as in previous studies [5, 13]. Motion‐corrupted frames were rejected, and frames were automatically cropped to avoid the influence of bright signals from adjacent organs. The brightest $b_{\mathrm{ref}}$ image was used as the fixed image for registration. STEAM datasets were registered using a rigid transformation, which functioned robustly for the intrinsically low‐SNR STEAM images. MCSE datasets used non‐rigid registration (itk‐Elastix [23]) to account for eddy‐current‐induced distortions. The *b*‐values of the STEAM dataset were corrected according to the actual Δ derived from the R‐R interval. The left‐ventricular (LV) myocardium was manually segmented using the initially estimated helix angle (HA) and mean diffusivity (MD) maps to avoid including border pixels containing blood pool signal. Misregistered frames were then manually excluded from the final diffusion tensor calculation (non‐linear least squares fitting). cDTI parameters, including MD, HA, fractional anisotropy (FA), and the absolute sheetlet angle (∣E2A∣), were obtained for each myocaridial voxel. Helix angle transmurality (HAT), representing the linear HA variation from endocardium to epicardium, was also derived.

Data quality analysis was assessed by comparing the median LV SNR, the percentage of pixels containing negative eigenvalues within the left ventricle, the Pearson $R^{2}$ of the linear fit of HAT and the standard deviation of the transverse angle (TA). The SNR was calculated for every pixel within the LV myocardium using a multiple repetition‐based approach [24]. It was defined as the ratio of the mean and the standard deviation of the signal intensity across the multiple repetitions of the same diffusion encoding directions and *b*‐value. For $b_{\text{high}}$, the SNR was further averaged over diffusion directions. Generally, a lower percentage of negative eigenvalues, an $R^{2}$ score closer to 1.0 and a lower standard deviation of TA indicate better cDTI data quality.

The HA map quality was visually assessed by a primary expert reader (14 years of experience in cDTI) under blinded conditions, with all identifying information (subject, sequence and cardiac phase) removed prior to evaluation. Scoring was based on the percentage of the myocardium exhibiting the expected transmural HV variation across the wall upon visual inspection; with a score of 0 for <50%, 1 for 50%–75%, 2 for 75%–95%, and 3 for >95% of the myocardium demonstrating the expected linear transmural HA variation in a healthy heart [5, 13]. Intra‐ and inter‐rater agreement were assessed using repeated readings by the same reader and an additional reader, and quantified using quadratically weighted Cohen's kappa.

Statistical analysis was conducted on the parameters and quality metrics across all three sequences for both cardiac phases. A normality test [25] was applied prior to analysis. For normally distributed datasets, results are presented as mean ± standard deviation, and repeated measures ANOVA [26, 27] was performed and followed by a pairwise *t*‐test when significance was observed. For non‐normally distributed data, results are presented as median [interquartile range], and the Friedman test [26, 28] was performed followed by the Wilcoxon signed‐rank test for pairwise comparisons. Bonferroni correction [29] was applied with an adjusted significance level of 0.05/3=0.017.

All datasets were included in the statistical analysis, including those of unsatisfactory quality that would normally be excluded in a clinical study, to fully reveal the effect of various sequences on the cDTI dataset quality.

#### 2.3.2 Study Protocol 2

DW datasets acquired using study protocol 2 were used to evaluate the motion‐related signal loss throughout diastasis. Therefore, motion‐corrupted frames were included in the post‐processing stage. Rigid registration was applied to the STEAM DW dataset. However, many of the MCSE datasets included low‐quality $b_{\text{high}}$ images with substantial motion‐induced signal loss, making registration unreliable. Therefore, landmark‐based affine registration (itk‐Elastix [23]) was applied to the MCSE DW datasets by placing six landmarks around the endocardium. For frames with no visible myocardium signal, landmarks were placed at the same positions as in the corresponding $b_{\mathrm{ref}}$ image within the same breath‐hold.

For each voxel in the LV myocardium, the apparent diffusion coefficient (ADC) was calculated in PRS directions, and the mean ADC (mADC) was derived by averaging across directions. TDs were expressed as a percentage of diastasis duration (e.g., 0%: beginning of diastasis; 50%: mid‐diastasis). Motion‐related signal loss was quantified as the percentage of LV myocardial pixels with ADC values exceeding the physiological limit of the self‐diffusivity of free water at $37^{\circ }C$ ($3\times 10^{-3}\;{\mathrm{mm}}^{2}/s$) [4, 30, 31].

## 3 Results

### 3.1 Study Protocol 1: cDTI at Peak Systole and Diastasis

**FIGURE 2:**
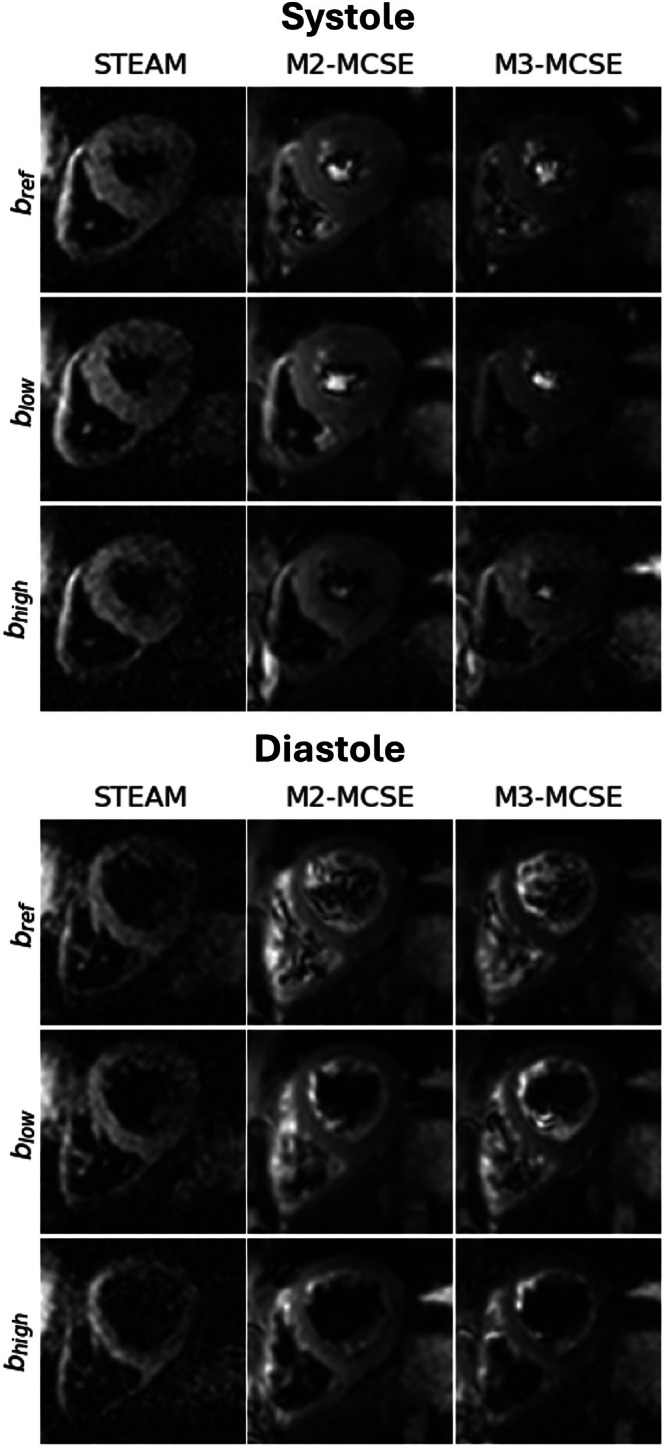
Example DW magnitude images from the systolic and diastolic cDTI dataset for the same subject. The $b_{\mathrm{ref}}$ image represents the *b*‐value of $∼34\;s/{\mathrm{mm}}^{2}$ for STEAM and $∼0\;s/{\mathrm{mm}}^{2}$ for M2‐ and M3‐MCSE, the $b_{\mathrm{low}}$ *b*‐value of $150\;s/{\mathrm{mm}}^{2}$ for STEAM and $50\;s/{\mathrm{mm}}^{2}$ for M2‐ and M3‐MCSE, and the $b_{\text{high}}$ represents the *b*‐value of $600\;s/{\mathrm{mm}}^{2}$ for STEAM and $500\;s/{\mathrm{mm}}^{2}$ for M2‐ and M3‐MCSE. The shown images were cropped around the heart region and normalized over the cropped area.

Representative $b_{\mathrm{ref}}$, $b_{\mathrm{low}}$ and $b_{\text{high}}$ images from a single repetition acquired during systole and diastole using STEAM, M2‐MCSE, and M3‐MCSE are shown in Figure 2. Residual blood pool signal was observed in M2‐MCSE and M3‐MCSE, particularly near the endocardium, but not in STEAM. Overall, cDTI acquisitions were successful, with systolic scans consistently demonstrating a lower percentage of rejected frames than diastolic scans across all methods (systole vs. diastole: 5.21% vs. 7.82% for STEAM; 2.51% vs. 8.96% for M2‐MCSE; 0.97% vs. 10.13% for M3‐MCSE). Some diastolic frames exhibited motion‐related signal loss (Figure S1) and were excluded from the cDTI parameter calculation. Fat‐related artifacts remained unavoidable in some cases for both cardiac phases (Figure S2).

**FIGURE 3:**
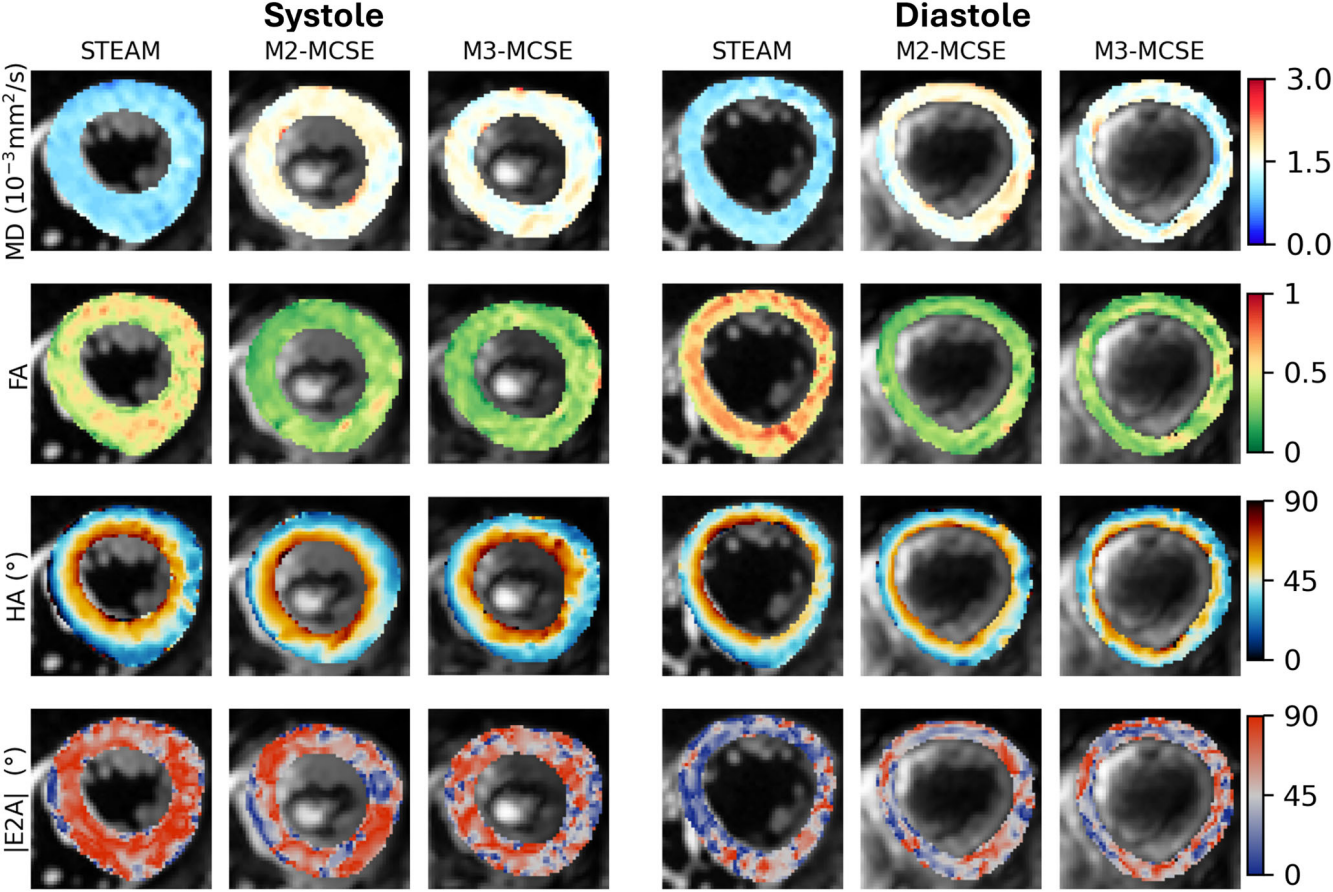
cDTI‐derived parametric maps for the subject shown in Figure 2: Mean diffusivity (MD), fractional anisotropy (FA), helix angle (HA) and absolute sheetlet angle (∣E2A∣) from the STEAM, M2‐MCSE and M3‐MCSE datasets.

The corresponding cDTI parametric maps for Figure 2 are shown in Figure 3. In this subject, all three sequences were able to generate high‐quality cDTI parametric maps, including MD, FA, HA, and ∣E2A∣. Other examples are provided in the Supporting Information (Figures S2 and S3). Overall, STEAM yielded lower MD and higher FA than both MCSE sequences. HA and FA maps from M3‐MCSE often appeared noisier than equivalent maps from M2‐MCSE data, with noise manifesting as random fluctuations, resulting in a grainier appearance.

**FIGURE 4:**
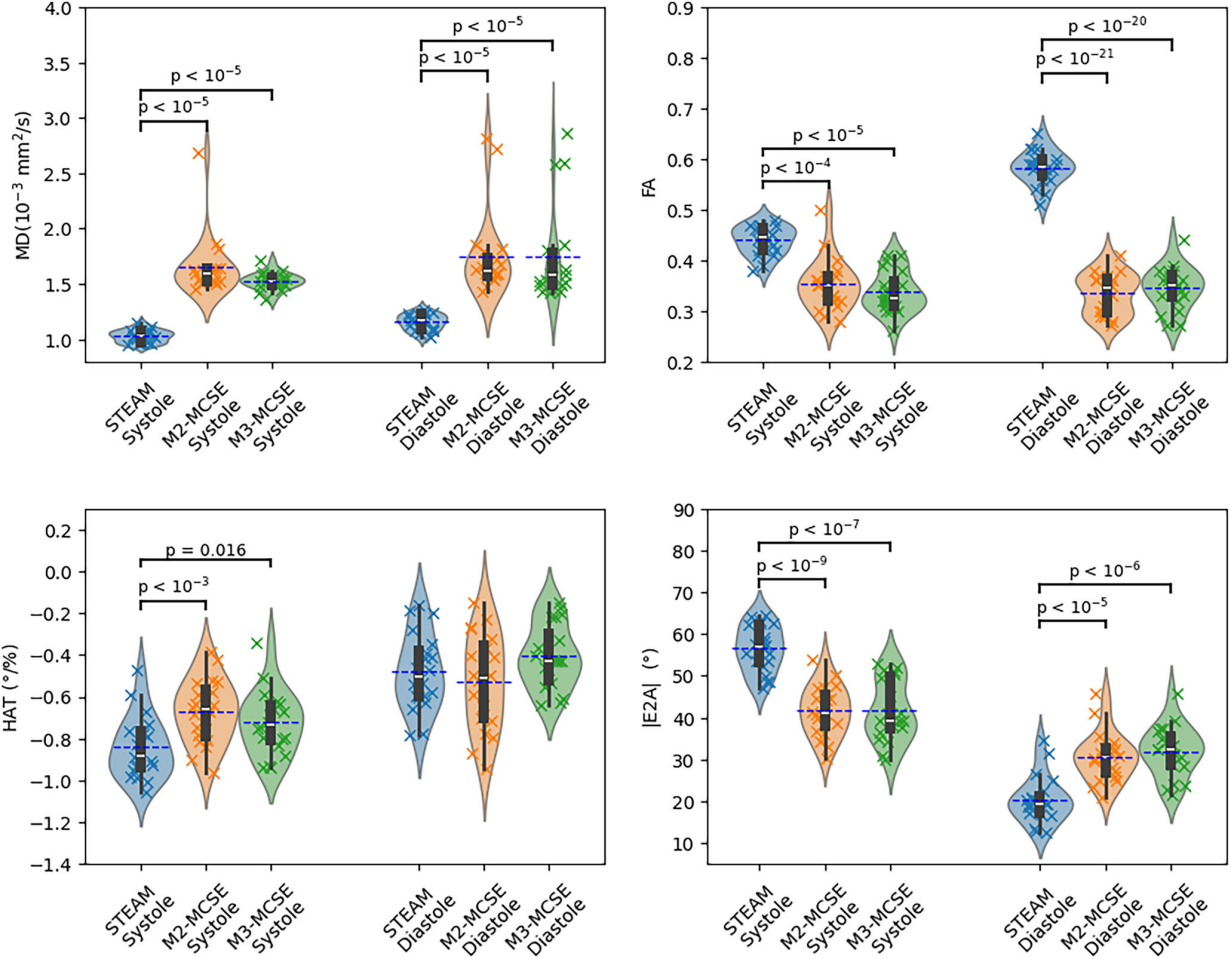
Violin plots demonstrate the median (white line), the mean (blue dashed line), and the interquartile range (IQR) of the cDTI‐derived parameters averaged over left‐ventricular myocardium. Parameters include mean diffusivity (MD), fractional anisotropy (FA), helix angle transmurality (HAT) from endocardium to epicardium, and the absolute sheetlet angle (∣E2A∣), STEAM, M2‐MCSE, and M3‐MCSE. Paired *t*‐tests were performed only when the overall group test indicated significant differences. Statistically significant pairwise comparisons are labeled with their corresponding *p* values.

The statistical comparison between the cDTI parameters is shown in Figure 4. The mean MD over the LV myocardium was significantly lower when obtained using STEAM compared to M2‐MCSE (STEAM: $1.03\left[0.11\right]\times 10^{-3}\;{\mathrm{mm}}^{2}/s$ vs. M2‐MCSE: $1.60\left[0.11\right]\times 10^{-3}{\mathrm{mm}}^{2}/s$, $p<10^{-5}$ for systole; STEAM: $1.17\left[0.13\right]\times 10^{-3}{\mathrm{mm}}^{2}/s$ vs. M2‐MCSE: $1.62\left[0.17\right]\times 10^{-3}{\mathrm{mm}}^{2}/s$, $p<10^{-5}$ for diastole) and M3‐MCSE (M3‐MCSE: $1.53\left[0.08\right]\times 10^{-3}{\mathrm{mm}}^{2}/s$, $p<10^{-5}$ for systole; M3‐MCSE: $1.59\left[0.30\right]\times 10^{-3}{\mathrm{mm}}^{2}/s$, $p<10^{-6}$ for diastole). No significant difference in MD was found between M2‐MCSE and M3‐MCSE in either systole (p=0.03) or diastole (p=0.43).

FA values obtained with STEAM were significantly higher than those obtained from either M2‐MCSE (STEAM: 0.45[0.04] vs. M2‐MCSE: 0.35[0.05], $p<10^{-4}$ for systole; STEAM: 0.59[0.03] vs. M2‐MCSE: 0.35[0.07], $p<10^{-21}$ for diastole) and M3‐MCSE (M3‐MCSE: 0.33[0.05], $p<10^{-5}$ for systole; M3‐MCSE: 0.35[0.04], $p<10^{-20}$ for diastole). However, there was no significant difference in FA between M2‐MCSE and M3‐MCSE (p=0.15 for systole, p=0.45 for diastole).

During systole, STEAM yielded significantly higher HAT magnitude ($-0.84\pm 0.15^{\circ }/\%$) compared to M2‐MCSE ($-0.67\pm 0.15^{\circ }/\%$, $p<10^{-3}$) and M3‐MCSE ($-0.72\pm 0.15^{\circ }/\%$, p=0.016). In diastole, repeated‐measures ANOVA indicated a significant difference among STEAM, M2‐MCSE and M3‐MCSE (STEAM: $-0.48\pm 0.18^{\circ }/\%$, M2‐MCSE: $-0.53\pm 0.23^{\circ }/\%$, M3‐MCSE: $-0.42\pm 0.15^{\circ }/\%$, p=0.03), although no pairwise comparisons reached significance after Bonferroni correction (p<0.017).

The ∣E2A∣ in systole obtained by M2‐MCSE and M3‐MCSE were significantly lower than those from STEAM (STEAM: 56.7±5.5°, M2‐MCSE: 41.7±6.1°, M3‐MCSE: 41.7±7.3°; $p<10^{-10}$ for M2‐MCSE, $p<10^{-9}$ for M3‐MCSE). In contrast, in diastole, the ∣E2A∣ observed for both M2‐MCSE and M3‐MCSE were significantly higher than with STEAM (STEAM: $20.4\pm 5.6^{\circ }$, M2‐MCSE: $30.6\pm 5.8^{\circ }$, M3‐MCSE: $31.9\pm 6.1^{\circ }$; $p<10^{-5}$ for M2‐MCSE, $p<10^{-6}$ for M3‐MCSE).

Overall, there was no significant difference in the parameters MD, FA, HAT and ∣E2A∣ between M2‐MCSE and M3‐MCSE in both systole and diastole. Detailed results in MD, FA, HAT and ∣E2A∣ were summarized in Table S3.

**FIGURE 5:**
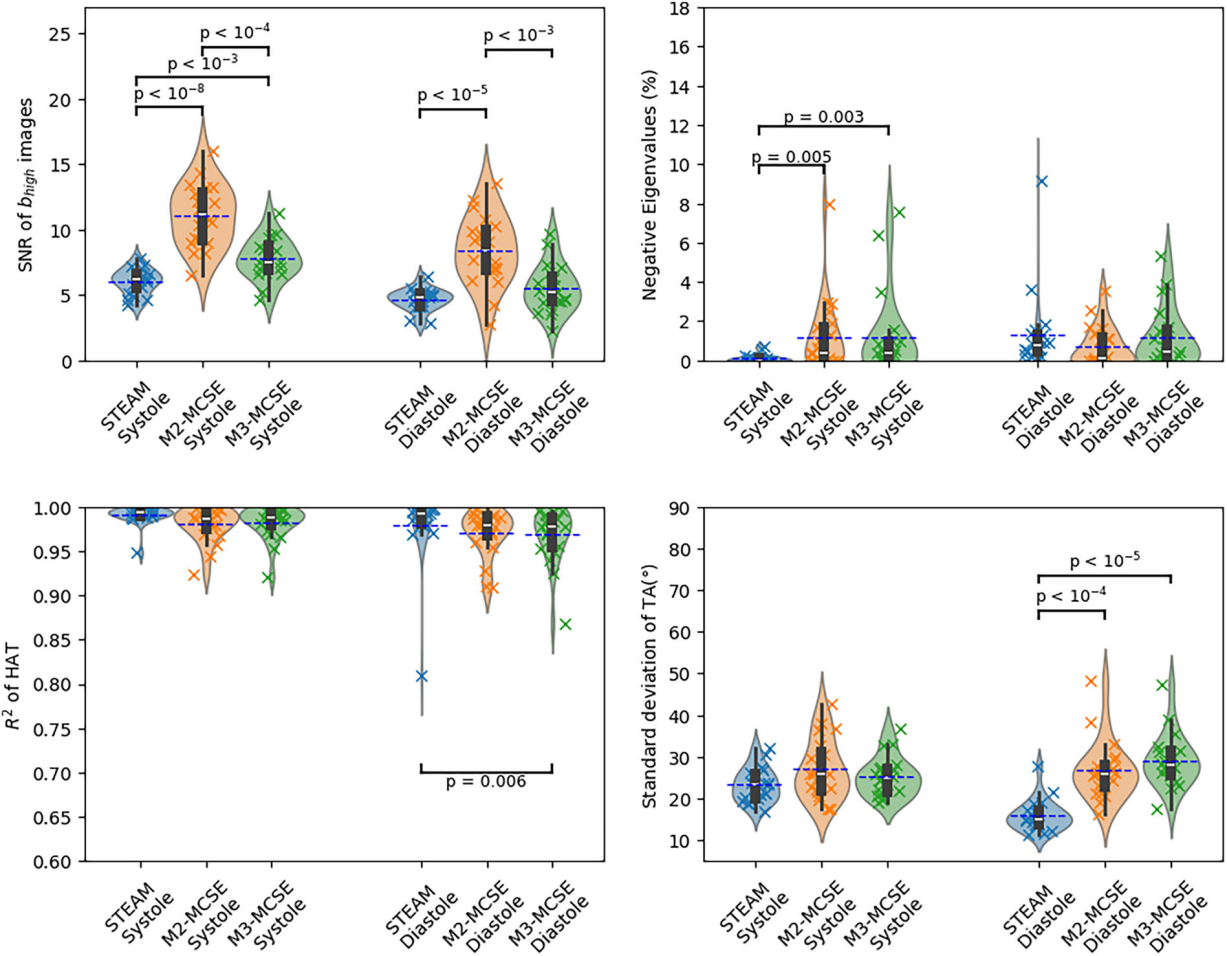
Violin plots demonstrate the median (white line), the mean (blue dashed line), and the interquartile range (IQR) of the cDTI data quality metrics, including the median of signal‐to‐noise ratio (SNR) of the high *b*‐value DW images, the percentage of negative eigenvalues over left‐ventricular myocardium, the $R^{2}$ score of the linear fitting of helix angle transmurality (HAT) and the standard deviation of transverse angle (TA) for STEAM, M2‐MCSE, and M3‐MCSE. Paired *t*‐tests were performed only when the overall group test indicated significant differences. Statistically significant pairwise comparisons are labeled with their corresponding *p* values.

The M2‐MCSE $b_{\text{high}}$ images had the highest SNR (Figure 5) for both systole (STEAM: 6.06±0.97, M2‐MCSE: 11.1±2.40, M3‐MCSE: 7.80±1.57, $p<10^{-8}$ compared to STEAM; $p<10^{-4}$ compared to M3‐MCSE) and diastole (STEAM: 4.67±0.82, M2‐MCSE: 8.41±2.69, M3‐MCSE: 5.54±1.83, $p<10^{-5}$ compared to STEAM; $p<10^{-3}$ compared to M3‐MCSE). While the diastolic $b_{\mathrm{ref}}$ SNR of M3‐MCSE showed no significant difference compared to STEAM, the systolic $b_{\mathrm{ref}}$ SNR of M3‐MCSE was significantly higher than that of STEAM. The SNR reduction in M3‐MCSE relative to M2‐MCSE was quantified as 74%±14% for systolic $b_{\mathrm{ref}}$ images and 70%±11% for diastolic $b_{\mathrm{ref}}$ images, and 71%±11% for systolic $b_{\text{high}}$ images and 66%±10% for diastolic $b_{\text{high}}$ images.

For systolic data, STEAM produced a significantly lower percentage of negative eigenvalues in the LV myocardium compared to M2‐MCSE (STEAM: 0[0.14]% vs. M2‐MCSE: 0.39[1.7]%, p=0.005) and M3‐MCSE (M3‐MCSE: 0.38[0.97]%, p=0.003). In the diastolic dataset, there was no overall significant difference between the three methods (STEAM: 0.77[0.85]%, M2‐MCSE: 0.14[1.18]%, M3‐MCSE: 0.43[1.57]%, p=0.38).

The Pearson $R^{2}$ score of the linear fit for HAT was high across all three methods in both systole and diastole (STEAM: 0.99[0.01] for systole, 0.99[0.02] for diastole, M2‐MCSE: 0.98[0.02] for systole, 0.98[0.02] for diastole, M3‐MCSE: 0.99[0.02] for systole, 0.98[0.03] for diastole). In systole, there were no significant differences between $R^{2}$ scores among the three methods, whereas diastolic STEAM had a significantly higher $R^{2}$ score compared to diastolic M3‐MCSE (p=0.006).

Additionally, for systolic cDTI, the standard deviation of TA showed a significant difference in the overall repeated measures ANOVA test (p=0.048) but no significant difference in all pairwise comparisons among the three methods (STEAM: $23.3\pm 4.1^{{}^{\circ}}$, M2‐MCSE: $27.0\pm 7.2^{{}^{\circ}}$, M3‐MCSE: $25.2\pm 4.8^{{}^{\circ}}$; all p>0.05; Bonferroni‐corrected threshold p<0.017). However, STEAM had a lower standard deviation of TA compared to M2‐MCSE and M3‐MCSE in diastole (STEAM:$15.0{\left[3.4\right]}^{{}^{\circ}}$, M2‐MCSE: $26.0{\left[5.5\right]}^{{}^{\circ}}$, M3‐MCSE: $27.9{\left[5.7\right]}^{{}^{\circ}}$; $p<10^{-4}$ for M2‐MCSE and $p<10^{-5}$ for M3‐MCSE). There was no difference in the standard deviation of TA in diastole between M2‐MCSE and M3‐MCSE (p=0.040).

**FIGURE 6:**
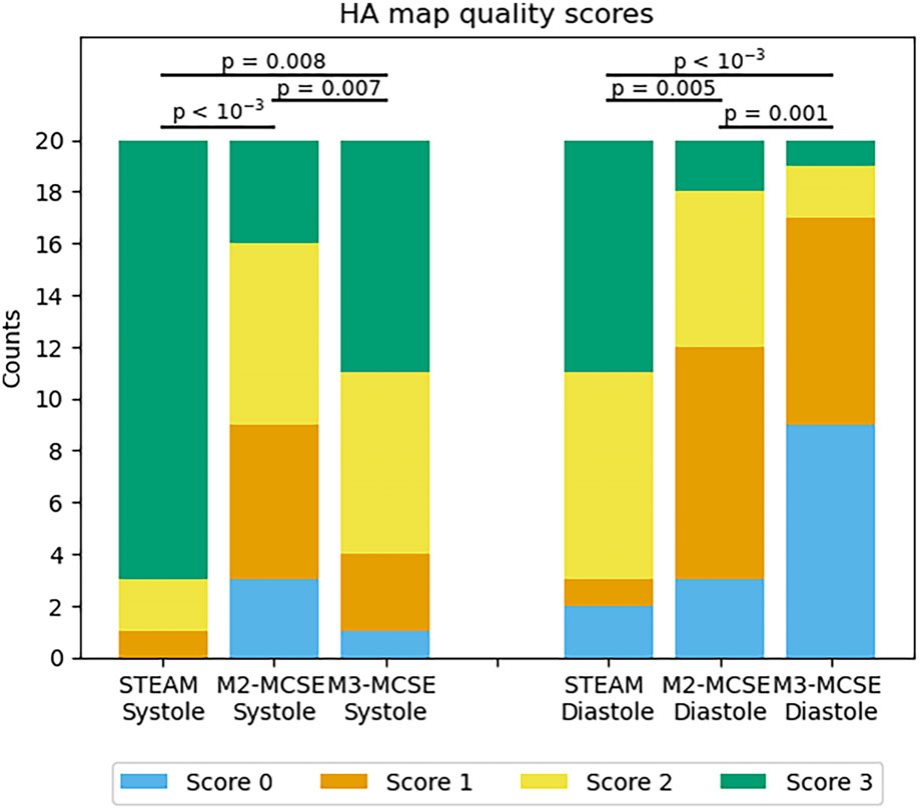
Comparison of helix angle (HA) map subjective quality scores across the STEAM, M2‐MCSE, and M3‐MCSE datasets acquired in both systole and diastole. The stacked bars illustrate the distribution of quality scores from 20 subjects, with statistical significance marked by *p* values positioned above the bars. Each dataset was scored blindly: A score of 0 indicates <50% of the LV myocardium with a normal transmural variation in HA, a score of 1 indicates between 50% and 75%, a score of 2 indicates between 75% and 95%, and a score of 3 represents >95%.

Inter‐ and intra‐rater agreement of the subjective HA map scoring ranged from good to excellent (κ = 0.77–0.90, Table S4). The HA map scoring (Figure 6) shows that the systolic HA map generally scored higher than the diastolic HA map (median HA map score for systole vs. diastole: STEAM: 3 vs. 2, M2‐MCSE: 2 vs. 1, M3‐MCSE: 2 vs. 1). In systole, all STEAM datasets scored higher than 0, and 1/20 M3‐MCSE dataset scored 0, whereas 3/20 M2‐MCSE datasets scored 0. STEAM had significantly higher HA map scores compared to M2‐MCSE ($p<10^{-3}$) and M3‐MCSE (p=0.008), and M3‐MCSE scored significantly higher than M2‐MCSE in systole (p=0.007). In diastole, a higher proportion of HA maps were rated 0 (2/20 for STEAM, 3/20 for M2‐MCSE and 9/20 for M3‐MCSE). STEAM also yielded significantly higher scores than M2‐MCSE and M3‐MCSE in diastole (p=0.005 for M2‐MCSE, $p<10^{-3}$ for M3‐MCSE). In contrast to the results in systole, the M3‐MCSE HA maps scored significantly lower than M2‐MCSE in diastole (p=0.001). Overall, these results demonstrate inconsistent HA map quality across sequences and cardiac phases.

### 3.2 Study Protocol 2: Evaluating Motion Robustness Throughout Diastasis

**FIGURE 7:**
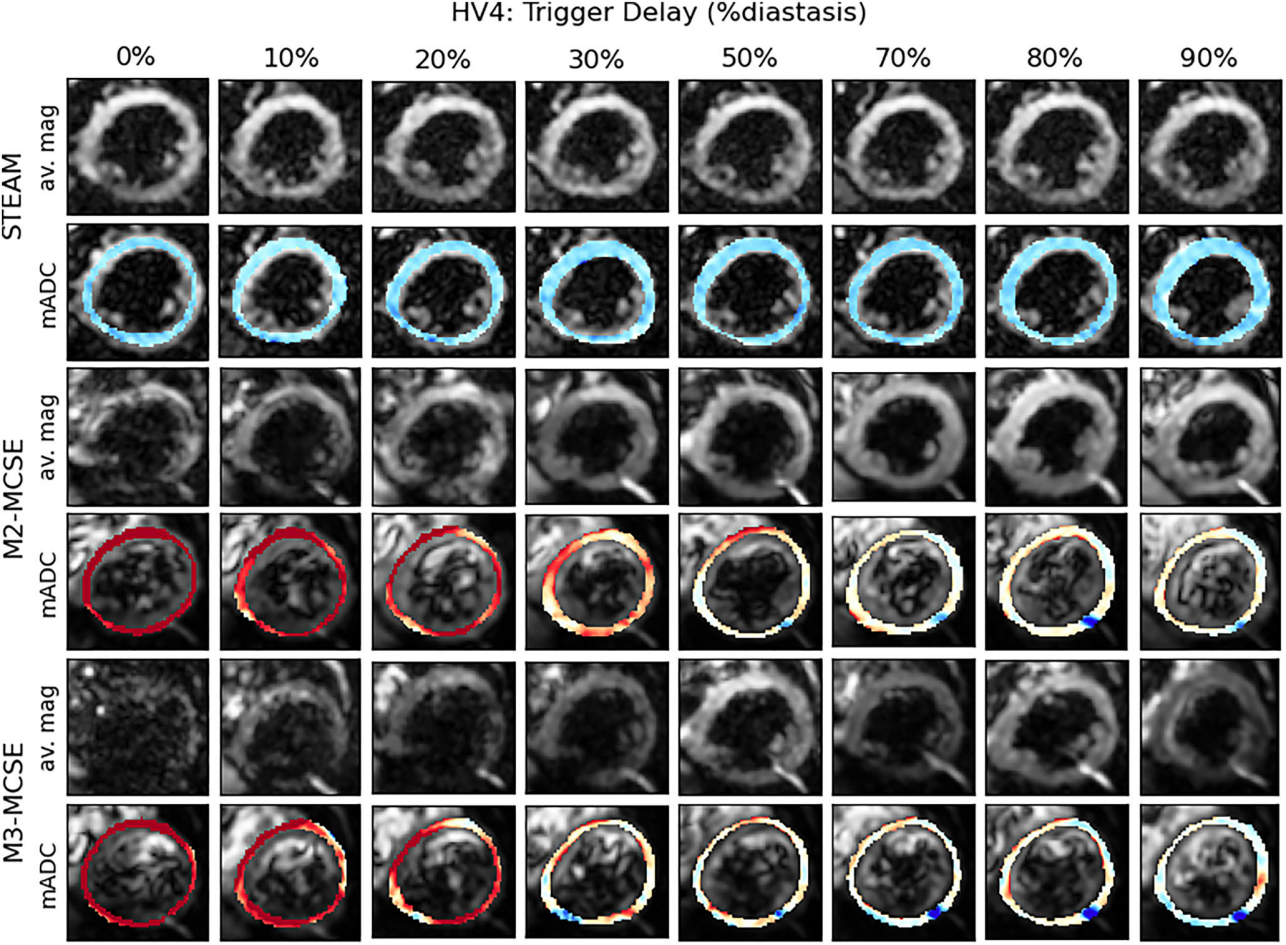
The averaged $b_{\text{high}}$ magnitude images across a single repetition of the three orthogonal diffusion encoding directions, alongside the corresponding mean apparent diffusion coefficient (mADC) for the segmented LV myocardium in one example subject. Trigger delays are expressed as a percentage of diastasis (%diastasis). A total of five healthy volunteers were imaged, with healthy volunteer 4 (HV4) shown here.

Figure 7 shows examples of average $b_{\text{high}}$ DW images (averaged from a single repetition of each PRS diffusion encoding direction) and mADC maps for TDs throughout diastasis. While STEAM produced DW images and mADC maps with consistent quality over all measured TDs, M2‐MCSE and M3‐MCSE exhibited severe motion‐related signal loss during early diastasis (0%∼20%). These DW images with visually evident motion‐related signal loss were included in the ADC calculation, which led to a substantial region of mADC exceeding $3\times 10^{-3}{\mathrm{mm}}^{2}/s$. Nevertheless, the motion‐related signal loss decreased markedly between 30% and 90% of diastasis.

**FIGURE 8:**
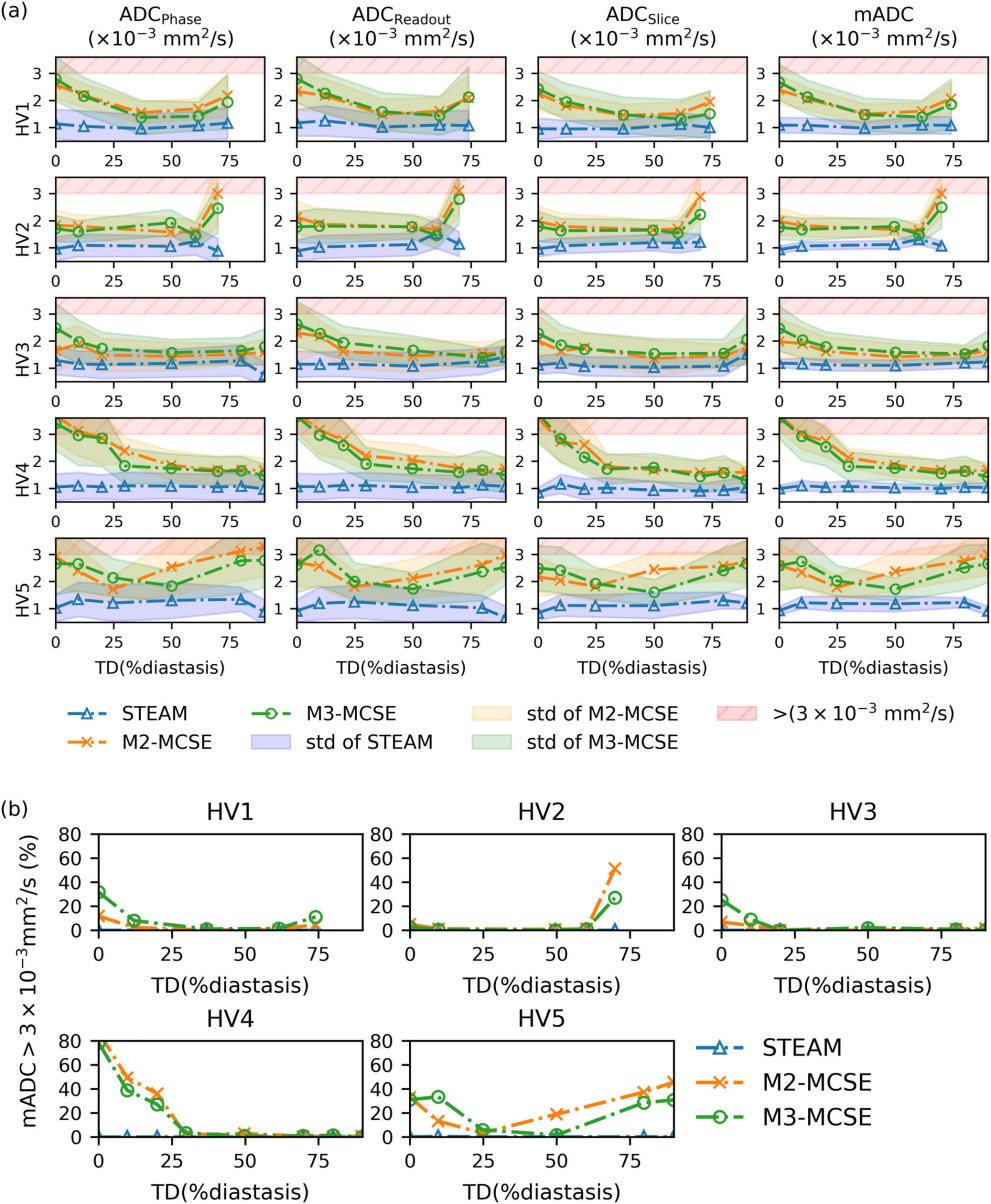
(a) Average apparent diffusion coefficient (ADC) across the left‐ventricular (LV) myocardium in each of the three orthogonal diffusion encoding directions and the mean ADC (mADC) for STEAM, M2‐MCSE, and M3‐MCSE against the percentage of diastasis for each of the 5 healthy volunteers imaged. Shaded regions show the corresponding standard deviation (std) of the ADC over the myocardium at each trigger delay (TD), and the red hatched region indicates where the ADC exceeds $3\times 10^{-3}{\mathrm{mm}}^{2}/s$ (approx. the ADC for unhindered diffusion at body temperature). (b) Percentage of LV pixels with a calculated mADC that exceeds the $3\times 10^{-3}{\mathrm{mm}}^{2}⁄s$ physiological limit.

Figure 8(a) shows average ADC and mADC across all five volunteers, where the ADCs for M2‐MCSE and M3‐MCSE were typically more likely to be elevated at the beginning and towards the end of diastasis, leading to the increased percentage of voxels with mADC exceeding $3\times 10^{-3}{\mathrm{mm}}^{2}/s$ (Figure 8(b)), whereas STEAM maintained relatively stable ADCs and had no voxels with the ADC exceeding this limit.

## 4 Discussion

This study evaluated the performance of STEAM, M2‐MCSE and M3‐MCSE cDTI sequences on a commercially available scanner with a UHP gradient system (200 mT/m, 200 T/m/s) at both systolic and diastolic time points, with an additional focus on robustness to cardiac motion throughout the diastolic rest phase.

STEAM demonstrated consistent performance in both systole and diastole with good‐quality cDTI datasets and only minor variations in mADC across diastasis, indicating a strong resistance to cardiac motion. As expected, MD increased, and FA decreased for M2‐ and M3‐MCSE compared with STEAM, in line with earlier comparison studies between STEAM and M2‐MCSE [5, 9], likely reflecting the different diffusion encoding mechanisms of the sequences. STEAM primarily encodes displacement decorrelation over longer diffusion time (one RR interval, ∼600–1000 ms), whereas spin echo sequences are more sensitive to velocity decorrelation over much shorter diffusion times, typically on the order of tens of milliseconds, depending on the gradient waveform design [32, 33]. However, STEAM is less compatible with true free‐breathing acquisitions and interleaved multislice imaging with a reduced phase FOV, which limits its broader applicability, whereas MCSE sequences are better suited [34, 35, 36].

The purpose of this study was to compare the novel M3‐MCSE method with established M2‐MCSE and STEAM to evaluate its potential for future clinical applications. The STEAM protocol (*b* = 150/600 s/mm^2^) followed previously optimized implementations applied in clinical research studies [37], ensuring optimal conditions. For MCSE, *b*‐values of 50–100/450–500 s/mm^2^ are commonly used [13, 14]. Therefore, a higher *b*‐value of 500 s/mm^2^ was selected to exploit the gradient performance of the current system. Consequently, the *b*‐values were not exactly matched. However, this did not hinder comparison, as additional experiments with different *b*‐value subsets yielded consistent trends in MD, FA, and ∣E2A∣ across sequences, with only minor variations in HAT, likely due to increased noise sensitivity when tensor fitting with fewer *b*‐values (Figures S4 and S5).

Compared with STEAM, M2‐MCSE exhibited greater sensitivity to cardiac phase, particularly in diastasis. However, the UHP gradient system improved the reliability of M2‐MCSE during diastole by shortening TE. Two previous studies with standard gradients reported a ∼50% failure rate (3 T, 43 mT/m, TE =76 ms) [5] and ∼60% failure rate (3 T, 60 mT/m, TE =62 ms) [13] for diastolic scans. The UHP gradient system used in this work reduced TE to 51 ms and achieved a failure rate of 15% for diastolic scans (determined by those where the HA map was scored 0), although careful selection of TD remains necessary to minimize motion‐related signal loss during diastasis.

The UHP gradient system also enabled, for the first time, assessment of the reliability of using M3‐MCSE for human in vivo diastolic cDTI. The anti‐symmetric trapezoidal constant gradient duration design is the only analytically derived third‐order motion‐compensated gradient waveform published to date [15], and would require a TE of approximately 98ms to run on a system with 60mT/m to reach a *b*‐value of $500\;s/{\mathrm{mm}}^{2}$. The UHP gradient system shortened TE for M3‐MCSE to 74ms for the same *b*‐value, making the in vivo M3‐MCSE feasible.

For both systole and diastasis, no statistical differences were found between M2‐MCSE and M3‐MCSE in key cDTI‐derived parameters such as MD, FA, HAT and ∣E2A∣. Afzali et al. reported higher MD values for systolic M3‐MCSE compared with M2‐MCSE [18]. This difference with the findings here could potentially be due to the difference in the diffusion gradient waveform design, as arbitrary gradient waveforms were employed in their study. There was also no statistically significant difference between the two MCSE methods in the cDTI quality metrics presented here, such as the percentage of negative eigenvalues, the Pearson $R^{2}$ score of HAT, and the standard deviation of TA.

Despite similar data quality metrics, M2‐MCSE and M3‐MCSE differ significantly in SNR. The longer motion‐compensated diffusion encoding gradient in M3‐MCSE theoretically provides additional motion compensation at a substantial SNR penalty due to the longer TE. Assuming a myocardial T2 of ∼46 ms at 3 T in healthy volunteers [38], the theoretical SNR ratio between M3‐MCSE and M2‐MCSE is approximately 61% $\left(\frac{S_{M_{3}}}{S_{M_{2}}}=e^{-\left(TE_{M_{3}}-TE_{M_{2}}\right)/T2_{\text{myocardium}}}=e^{-\left(74\mathrm{ms}-51\mathrm{ms}\right)/46\mathrm{ms}}=61\%\right)$. The measured ratio for $b_{\mathrm{ref}}$ images (74%±14% for systole, 70%±11% for diastole) and $b_{\text{high}}$ images (71%±11% for systole, 66%±10% for diastole) was slightly higher, but within one standard deviation of the theoretical value.

In systole, M3‐MCSE demonstrated improved robustness compared to M2‐MCSE. It achieved significantly higher HA map scores than those with M2‐MCSE, as well as exhibited a lower failure rate (1/20 vs. 3/20 for M2) and a higher proportion of high‐quality HA maps (score 3: 9/20 vs. 4/20), suggesting third‐order motion compensation provides additional benefit and stability during systole.

Conversely, during diastole, M3‐MCSE did not confer a robustness advantage. The failure rate was higher for M3‐MCSE (9/20) compared to M2‐MCSE (3/20). M2‐MCSE yielded higher HA map quality scores than M3‐MCSE, indicating that the higher‐order motion‐compensation provided by the current M3‐MCSE sequence did not translate to improved image quality in diastolic cardiac phases. Neither MCSE method achieved a diastolic HA map quality comparable to STEAM, where the best quality HA maps (scored 3) were obtained 11/20 datasets, compared to only 2/20 M2‐MCSE and 1/20 M3‐MCSE datasets.

The reduced diastolic performance of M3‐MCSE, particularly in generating meaningful HA maps during diastole, can be attributed to several factors. First, its lower SNR compared to M2‐MCSE, making HA estimation more vulnerable to noise‐induced error, particularly as the myocardial wall is thinner during diastole and more susceptible to the partial‐volume effect. Additional analysis using non‐local means denoising suggested that the reduced SNR due to longer TE of M3‐MCSE contributes to its diastolic performance, although it does not fully account for it (Figure S6). These results indicate that implementing M3‐MCSE with shorter TE may substantially improve its performance and clinical utility.

Another factor may be the longer diffusion encoding gradient required for third‐order motion compensation. Although higher‐order motion compensation aims to recover more motion‐related signal loss, the longer encoding duration may increase the likelihood of involving more complex cardiac motion. Such motion may not be fully compensated, particularly in diastasis, where motion may be of lower amplitude than during systolic contraction but may also be more complex [3].

TDs in this study were selected to optimize M2‐MCSE data quality, assuming this sequence would be most sensitive to timing. ADC analysis across multiple diastolic TDs showed that both M2‐MCSE and M3‐MCSE exhibited strong dependence on the TD selection, and shared a similar and narrow subject‐specific optimal range in diastasis. Notably, no clear expansion of the feasible diastolic TD window was observed for M3‐MCSE compared to M2‐MCSE, indicating that its poorer performance in diastole was not primarily due to matching the M3‐MCSE timing to that optimized for M2‐MCSE, and both sequences require careful selection of TD when acquired during diastole.

To further evaluate whether the observed differences could arise from sequence‐dependent factors such as eddy currents rather than motion effects, additional motion‐free phantom experiments were conducted. All sequences did not show clear sequence‐dependent bias in MD or FA (Figure S7), again suggesting that motion could be the primary factor for poor M3‐MCSE dataset quality.

Overall, M3‐MCSE demonstrated robust systolic performance with a modest advantage over M2‐MCSE in terms of generating higher quality systolic HA maps, despite its longer TE. While M2‐MCSE remains sufficient for most systolic acquisitions [5, 9, 11, 13, 14], higher‐order motion compensation may provide additional benefits when the gradient performance permits for systolic acquisition. However, the advantages of improved motion compensation did not necessarily overcome the drawbacks from reduced SNR and extended motion‐sensitive duration and could not translate into broader robustness across diastasis.

There are several technical limitations affecting MCSE performance. These include incomplete suppression of epicardial and subcutaneous fat with a chemical‐shift fat‐suppression pulse. We modified the PE direction for each subject to shift the fat artifact away from the myocardium, where possible. Nonetheless, residual fat signal was present in the LV myocardium in some cases (18 out of 80 MCSE datasets), resulting in unrealistically low MD, high FA, high percentages of negative eigenvalues and distorted HA maps at the position of these artifacts. However, the fat artifacts were consistent between M2‐MCSE and M3‐MCSE for comparison purposes. Alternative fat suppression methods, such as water excitation or spectral attenuated inversion recovery (SPAIR), could be implemented to address this issue in future [39].

Another limitation is that only a single mid‐ventricular slice was acquired due to scan time constraints (∼75 min/session). There might be a performance difference between M2 and M3 across different slices, and extending the acquisition to multiple short axes to enable American Heart Association (AHA) segment‐based analysis would be valuable and is an important direction for future work.

Although the UHP gradient system provides great advantages in reducing the diffusion encoding gradient duration, the maximum gradient amplitude available was not reached for the MCSE sequences used in this study, predominantly due to cardiac nerve stimulation. For the *b*‐value of $500s/{\mathrm{mm}}^{2}$, the maximum gradient reached 123mT/m for M2‐MCSE and 144mT/m for M3‐MCSE. One potential method to make more efficient use of the available gradient strength is to use arbitrary (otherwise known as “free”) asymmetric gradient waveform design [18, 40, 41, 42], maximizing the achievable gradient strength while having the potential to incorporate the cardiac nerve stimulation model as one of the constraints in the encoding gradient design, resulting in higher *b*‐values for shorter encoding durations. It provides additional advantages such as higher diffusion encoding efficiency, further reducing the TE.

A basic 6‐direction diffusion encoding scheme was used in this study. It is the minimum requirement for tensor estimation and is commonly applied for many cDTI studies. Alternative diffusion encoding schemes may allow higher gradient amplitudes per axis without exceeding nerve stimulation limits, albeit not necessarily resulting in higher *b*‐values. Increasing the number of diffusion encoding directions has been previously shown to improve the precision and accuracy of the cDTI metrics [43]. While the use of the minimum 6 diffusion encoding directions did not hinder this comparison study between the 3 diffusion encoding schemes, and the findings are translatable to protocols with higher numbers of diffusion encoding directions, further optimization of the diffusion encoding scheme will be investigated in future studies.

## 5 Conclusion

We compared STEAM, M2‐MCSE, and M3‐MCSE sequences for both systolic and diastolic cDTI, focusing on the feasibility of using a clinical MRI scanner with UHP gradients for higher‐order motion compensation. The high‐gradient performance improved the diastolic reliability of M2‐MCSE compared with previous studies using more modest‐gradient hardware, and for the first time, we evaluated M3‐MCSE in diastole.

M3‐MCSE achieved higher HA map quality than M2‐MCSE in systole, suggesting that third‐order motion compensation can still be beneficial despite the SNR loss due to prolonged TE. In diastole, the derived cDTI parameters and quality metrics are broadly comparable between M2‐MCSE and M3‐MCSE. However, the SNR reduction and residual cardiac motion affected M3‐MCSE more severely for diastolic imaging. Both M2‐MCSE and M3‐MCSE may achieve optimal diastolic quality when TDs are carefully selected in diastole. However, STEAM remains the method of choice for robust imaging in diastole.

Overall, M3‐MCSE is a feasible alternative technique for systolic cDTI on a UHP gradient clinical system, potentially more robust. Future research into more advanced motion compensation gradient waveform designs, such as arbitrary gradient waveform designs, represents a promising avenue for further improving MCSE robustness across the cardiac cycle and enhancing its clinical applicability.

## Funding

British Heart Foundation Programme Grant RG/19/1/34160; Engineering and Physical Science Research Council (EPSRC), Centre for Doctoral Training in Smart Medical Imaging, co‐funded by Siemens Healthineers EP/S022104/1; Chan Zuckerberg Initiative DAF2021‐000000.

## Conflicts of Interest

Ke Wen declares that Siemens Healthcare partly funds their PhD project. Dudley J. Pennell, Andrew D. Scott and Sonia Nielles‐Vallespin declare departmental research support from Siemens Healthcare. Karl P. Kunze is an employee of Siemens Healthcare Limited.

## Supporting information


**Table S1:** Subject demographics.
**Table S2:** Diffusion encoding gradient parameters for the STEAM, M2‐MCSE, and M3‐MCSE sequences, including ramp‐up time, flat‐top duration, ramp‐down time and corresponding effective slew rate.
**Table S3:** A summary of cDTI‐derived parameters including mean diffusivity (MD), fractional anisotropy (FA), helix angle transmurality (HAT) and the absolute sheetlet angle (∣E2A∣). Data that was compared using the Wilcoxon signed‐rank test is shown as median[interquartile range], data that was compared using the pairwise *t*‐test is shown as mean ± standard deviation.
**Table S4:** Intra‐rater and inter‐rater agreement for HA map quality score.
**Figure S1:** Example of motion‐corrupted frames from one healthy volunteer.
**Figure S2:** Example of a cDTI dataset where the quality suffers due to residual fat signal artifacts. The $b_{r}\mathrm{ef}$ images are displayed with an intensity window of [0, 0.5] to enhance the visualization of these fat artifacts. Note that the STEAM sequence does not suffer from these fat artifacts as a consequence of the long mixing time.
**Figure S3:** Additional examples of a cDTI dataset showing diffusion‐weighted images, MD, FA, HA and ∣E2A∣ with different HA map scores: 0 (s0), 1 (s1), 2 (s2), 3 (s3).
**Figure S4:** Subset cDTI analysis with only b_low_ and b_high_ images showing the violin plots comparison of MD, FA, HAT and ∣E2A∣.
**Figure S5:** Subset cDTI analysis with only b_ref_ and b_high_ images showing the violin plots comparison of MD, FA, HAT and ∣E2A∣.
**Figure S6:** Comparison of the HA map quality score derived from the denoised M3‐MCSE dataset. The statistical comparison was conducted between STEAM vs. denoised M3‐MCSE, M2‐MCSE vs. denoised M3‐MCSE and M3‐MCSE vs. denoised M3‐MCSE for both systole and diastole.
**Figure S7:** Coefficient of variation maps (Cov) calculated across all the directions for $b_{\text{high}}$ images, MD maps, and FA maps generated from the cDTI scans on a stationary phantom for M2‐MCSE, M3‐MCSE, STEAM and monopolar spin echo sequences. Mean and standard deviation (std) of metrics are labeled on top of each map.

## Data Availability

The data that support the findings of this study are available on request from the corresponding author. The data are not publicly available due to privacy or ethical restrictions.
