# Supplementary material for: Evaluation of Third‐Order Motion‐Compensated Cardiac Diffusion Tensor Imaging Across Cardiac Phases Using an Ultra‐High‐Performance Clinical Scanner
Source: Magn Reson Med. 2026 Apr 22;96(3):1365–78. doi: 10.1002/mrm.70395 (PMC13327481; doi:10.1002/mrm.70395)
Supplement: Supplementary file 1 — Table S1: Subject demographics. Table S2: Diffusion encoding gradient parameters for the STEAM, M2‐MCSE, and M3‐MCSE sequences, including ramp‐up time, flat‐top duration, ramp‐down time and corresponding effective slew rate. Table S3: A summary of cDTI‐derived parameters including mean diffusivity (MD), fractional anisotropy (FA), helix angle transmurality (HAT) and the absolute sheetlet angle (∣E2A∣). Data that was compared using the Wilcoxon signed‐rank test is shown as median[interquartile range], data that was compared using the pairwise t‐test is shown as mean ± standard deviation. Table S4: Intra‐rater and inter‐rater agreement for HA map quality score. Figure S1: Example of motion‐corrupted frames from one healthy volunteer. Figure S2: Example of a cDTI dataset where the quality suffers due to residual fat signal artifacts. The bref images are displayed with an intensity window of [0, 0.5] to enhance the visualization of these fat artifacts. Note that the STEAM sequence does not suffer from these fat artifacts as a consequence of the long mixing time. Figure S3: Additional examples of a cDTI dataset showing diffusion‐weighted images, MD, FA, HA and ∣E2A∣ with different HA map scores: 0 (s0), 1 (s1), 2 (s2), 3 (s3). Figure S4: Subset cDTI analysis with only blow and bhigh images showing the violin plots comparison of MD, FA, HAT and ∣E2A∣. Figure S5: Subset cDTI analysis with only bref and bhigh images showing the violin plots comparison of MD, FA, HAT and ∣E2A∣. Figure S6: Comparison of the HA map quality score derived from the denoised M3‐MCSE dataset. The statistical comparison was conducted between STEAM vs. denoised M3‐MCSE, M2‐MCSE vs. denoised M3‐MCSE and M3‐MCSE vs. denoised M3‐MCSE for both systole and diastole. Figure S7: Coefficient of variation maps (Cov) calculated across all the directions for bhigh images, MD maps, and FA maps generated from the cDTI scans on a stationary phantom for M2‐MCSE, M3‐MCSE, STEAM and monopolar spin echo sequences. Mea [file MRM-96-1365-s001.pdf]

Supplementary Material

Supplementary Table S1 : Subject demographics

**Study Protocol**	**1**	**2**
Female, n (%)	12 (60%)	3 (60%)
Male, n (%)	8 (40%)	2 (40%)
Age (years, mean ± SD)	35 ± 9	24 ± 3

SD: standard deviation.

Supplementary Table S2 : Diffusion encoding gradient parameters for the STEAM, M2-MCSE, and M3-MCSE sequences, including ramp-up time, flat-top duration, ramp-down time and corresponding effective slew rate.

**Sequence**	**Amplitude [mT/m]**	**Ramp-up [µs]**	**Flat-top [µs]**	**Ramp-down [µs]**	**Effective Slew Rate [T/m/s]**
STEAM	28.54	1100	1170	1100	25.95
M2-MCSE (G1)	122.79	3280	0	3280	37.44
M2-MCSE (G2)	122.79	3280	3280	3280	37.44
M3-MCSE (G1)	51.23	4010	1070	4010	12.77
M3-MCSE (G2)	144.36	4010	1070	4010	35.99
M3-MCSE (G3)	92.13	4010	1070	4010	22.97

Supplementary Table S3 : A summary of cDTI-derived parameters including mean diffusivity (MD), fractional anisotropy (FA), helix angle transmurality (HAT) and the absolute sheetlet angle (|E2A|). Data that was compared using the Wilcoxon signed-rank test is shown as median[interquartile range], data that was compared using the pairwise t-test is shown as mean±standard deviation.

	Systole			Diastole		
	STEAM	M2-MCSE	M3-MCSE	STEAM	M2-MCSE	M3-MCSE
MD($\times 10^{-3}$mm^2/s)	1.03[0.11]	1.60[0.11]	1.53[0.08]	1.17[0.13]	1.62[0.17]	1.59[0.30]
FA	0.45[0.04]	0.35[0.05]	0.33[0.05]	0.59[0.03]	0.35[0.07]	0.35[0.04]
HAT (°/%)	-0.84 ± 0.15	-0.67 ± 0.15	-0.72 ± 0.15	-0.48 ± 0.18	-0.53 ± 0.23	-0.42 ± 0.15
|E2A| (°)	56.7 ± 5.5	41.7 ± 6.1	41.7 ± 7.3	20.4 ± 5.6	30.6 ± 5.8	31.9 ± 6.1

Supplementary Table S4 : Intra-rater and inter-rater agreement for HA map quality score.

Type	Comparison	Quadratic weighted κ
Intra-rater	Reader A-1 vs Reader A-2	0.89
Intra-rater	Reader B-1 vs Reader B-2	0.83
Inter-rater	Reader A-1 vs Reader B-1	0.90
Inter-rater	Reader A-2 vs Reader B-2	0.77

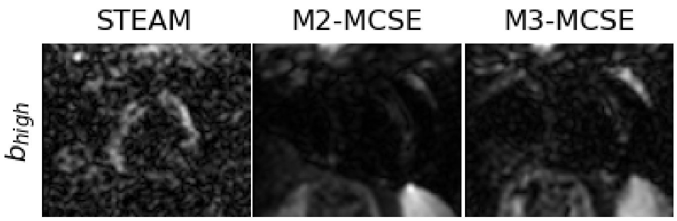

Supplementary Figure S1 : Example of motion-corrupted frames from one healthy volunteer.

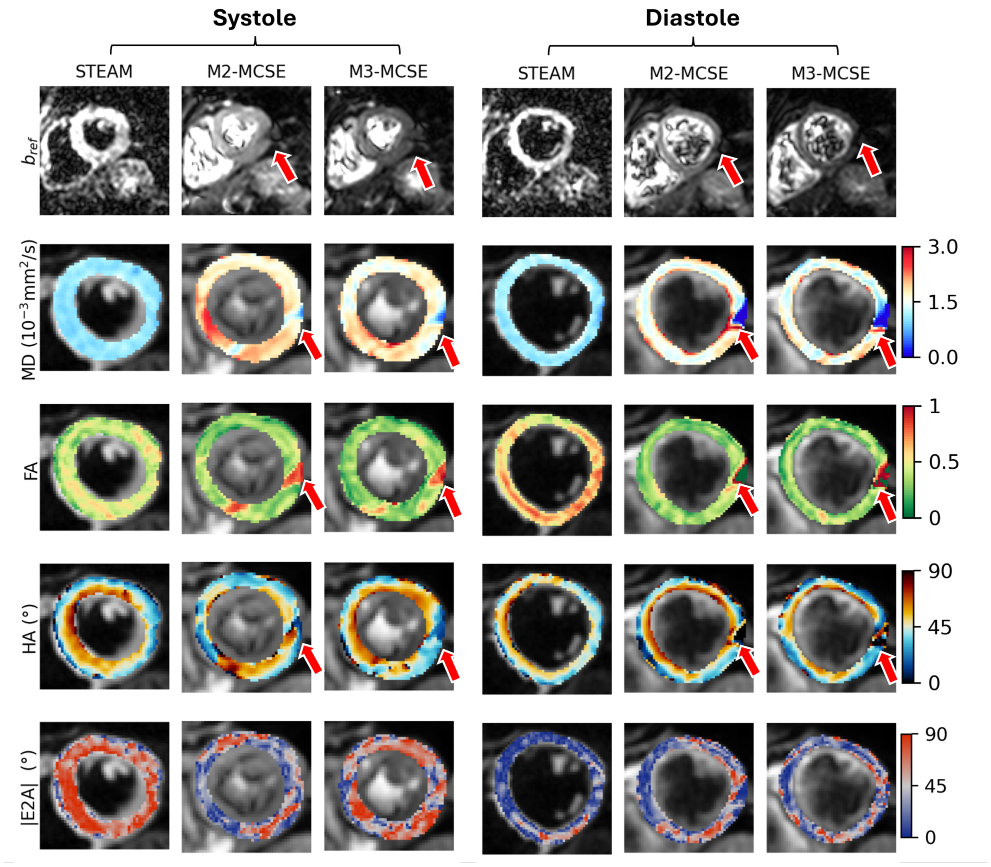

Supplementary Figure S2 : Example of a cDTI dataset where the quality suffers due to residual fat signal artefacts. The b_ref images are displayed with an intensity window of [0, 0.5] to enhance the visualisation of these fat artefacts. Note that the STEAM sequence does not suffer from these fat artefacts as a consequence of the long mixing time.

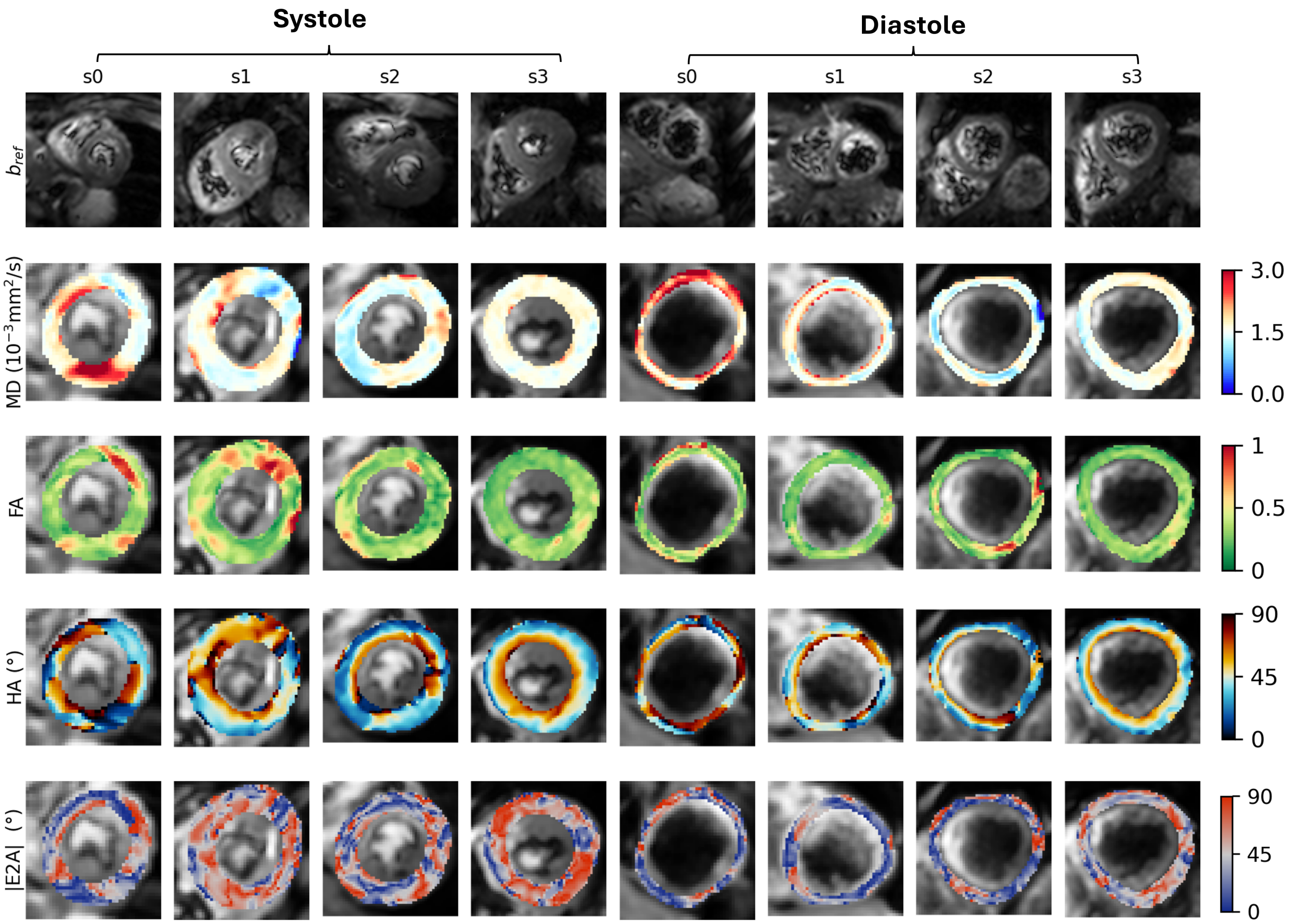

Supplementary Figure S3 : Additional examples of a cDTI dataset showing diffusion-weighted images, MD, FA, HA and |E2A| with different HA map scores:0 (s0), 1 (s1), 2 (s2), 3 (s3).

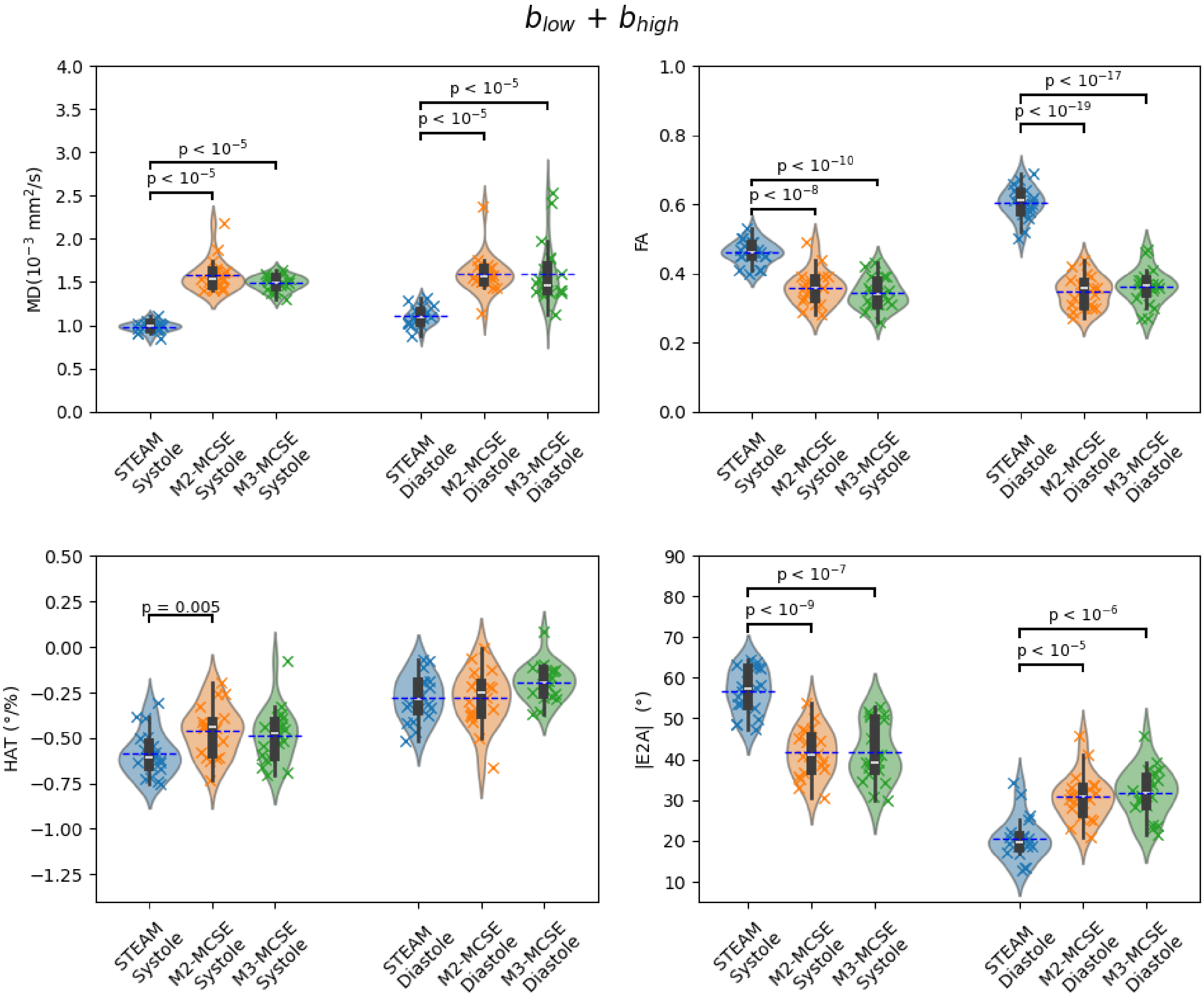

Supplementary Figure S4 : Subset cDTI analysis with only b_{low} and b_{high} images showing the violin plots comparison of MD, FA, HAT and |E2A|.

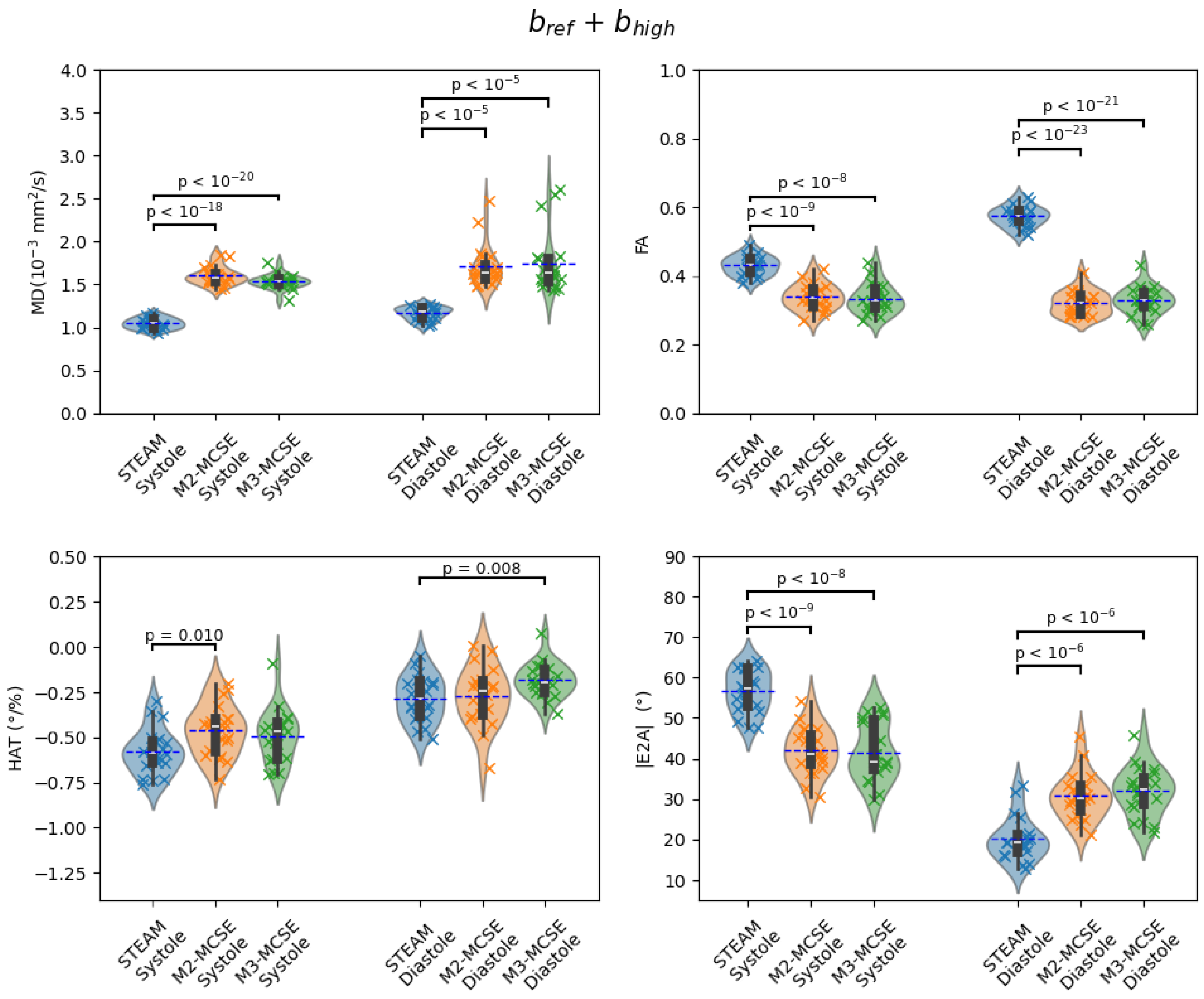

Supplementary Figure S5 : Subset cDTI analysis with only b_{ref} and b_{high} images showing the violin plots comparison of MD, FA, HAT and |E2A| .

The intrinsic SNR penalty due to longer TE for M3-MCSE was one of the confounding factors affecting the performance of M3-MCSE, particularly during diastole. The standard non-local means (NLM) approach (Buades et al., 2005, DOI: 10.1109/CVPR.2005.38) was applied to denoise all M3-MCSE diffusion-weighted images before tensor calculation. HA maps generated from the denoised M3-MCSE dataset were then scored following the same blinded assessment protocol as described in the original manuscript, with denoised and original results randomly interleaved. The results indicate that denoised M3-MCSE had an improved HA map quality in diastole but not in systole. The relative performance ranking of the methods remained consistent, indicating that a reduced SNR originating from a longer TE contributed to the performance of M3-MCSE for the diastolic scan, although it was not the sole factor involved. These results indicate that an implementation of M3-MCSE with shorter TE may substantially improve its performance and clinical utility.

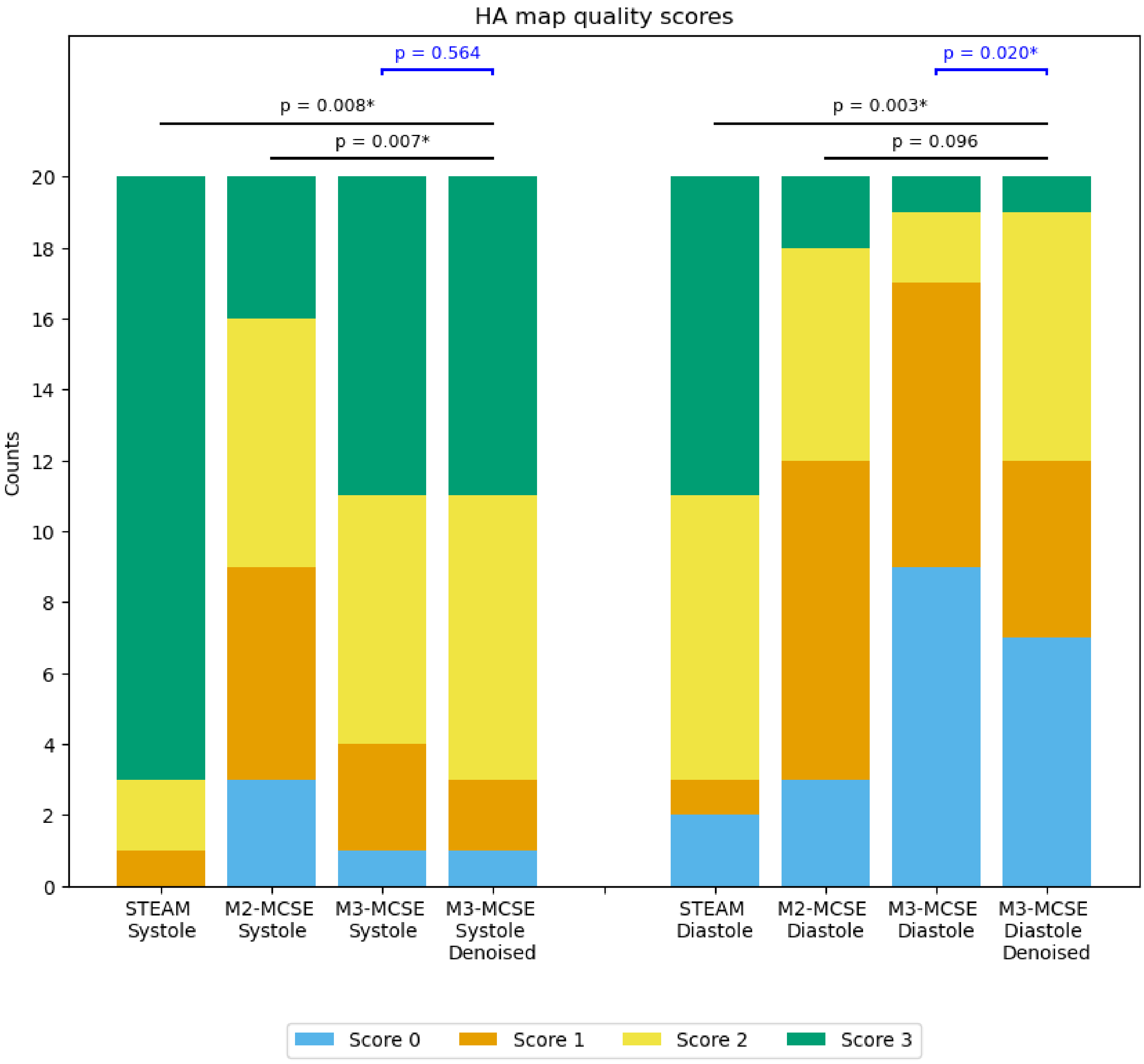

Supplementary Figure S6 : Comparison of the HA map quality score derived from the denoised M3-MCSE dataset. The statistical comparison was conducted between STEAM vs denoised M3-MCSE, M2-MCSE vs denoised M3-MCSE and M3-MCSE vs denoised M3-MCSE for both systole and diastole.

M2-MCSE, M3-MCSE and STEAM sequences were performed following the same protocol as in Study Protocol 1 on a stationary in vitro phantom without a microscale structure organisation to disentangle the effect of motion from all confounding factors, as well as to evaluate the effect of eddy current. An additional monopolar spin echo sequence was also performed following the same b-value schemes as M2-MCSE and M3-MCSE with a TE of 30ms as a reference measurement.

Eddy currents cause different geometric distortions in various diffusion encoding directions, which is the main reason for pixel-wise mismatches between the DW images at different directions under motion-free conditions. The distortion was quantified by calculating the pixel-wise coefficient of variation (CoV) (Aliotta et al., 2018, DIO: 10.1002/mrm.26709) across diffusion encoding directions for the b_{high} images. Eddy currents introduce different geometric distortions in various diffusion encoding directions, which lead to pixel-wise mismatches between the DW images acquired with different directions under motion-free conditions. These distortions were quantified by calculating the pixel-wise coefficient of variation (CoV) across diffusion encoding directions for the bhigh images (Aliotta et al., 2018, DOI: 10.1002/mrm. 26709). Phantom without microscale structure reduces the diffusion time-dependent effect on cDTI-derived metrics such as MD and FA. MD and FA maps were generated for all four sequences to evaluate their performance under motion-free conditions.

All sequences experienced the eddy-current effect with M2-MCSE and monopolar SE to be most affected. Nevertheless, the mean FA and MD values for all sequences remained comparable and within one standard deviation of each other.

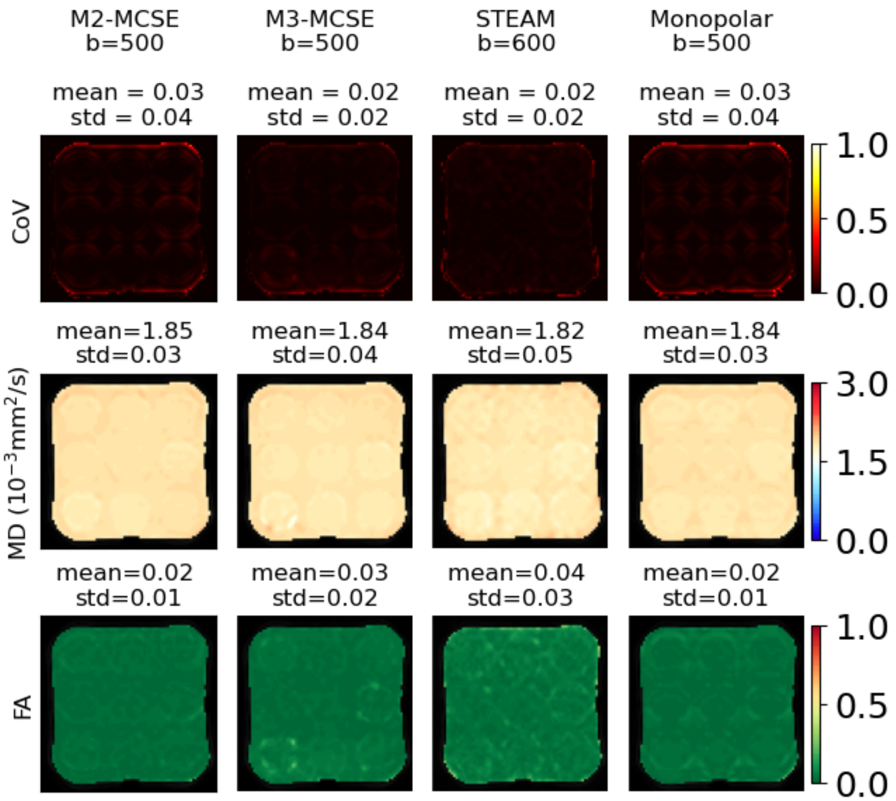

Supplementary Figure S7 : Coefficient of variation maps (Cov) calculated across all the directions for b_{high} images, MD maps, and FA maps generated from the cDTI scans on a stationary phantom for M2-MCSE, M3-MCSE, STEAM and monopolar spin echo sequences. Mean and standard deviation (std) of metrics are labelled on top of each map.
